# Supplementary material for: A Likelihood Approach for Real-Time Calibration of Stochastic Compartmental Epidemic Models
Source: PLoS Comput Biol. 2017 Jan 17;13(1):e1005257. doi: 10.1371/journal.pcbi.1005257 (PMC5240920; doi:10.1371/journal.pcbi.1005257)
Supplement: S1 File — (TAR.GZ) [file pcbi.1005257.s014.tar.gz › HSPH_Online-SI-Revision/Additions/PF09/n100-extreme-SEITR_noisy/n100-extreme-SEITR_noisy_PF_table-4.pdf]

1	R0	{1.38654, 9.85565}	{2.06451, 20.}	{{2.06301, 20.}, {2.0657, 20.}}	{48.8968, 102.929}	inf	4942.	6505.13	{6487.76, 6518.37}	14.7204	0.0691558	newly	7087.2	2573.5	{2519., 2631.}	63.688	0.440012	Reff	1.09645	1.70622	{1.70481, 1.7076}	55.6132	ar	92 379.	87 310.4	{86 921., 87 704.}	5.4868
2	R0	{2.36629, 12.2029}	{2.96942, 19.9833}	{{3., 20.}, {3., 20.}}	{25.5553, 63.7584}	inf	1894.	3314.27	{2669.89, 3554.38}	22.6319	0.0895086	newly	3034.	1614.12	{1335., 1787.}	46.7989	0.278372	Reff	2.22479	2.82841	{2.83692, 2.8633}	27.1356	ar	99 022.	95 609.5	{95 336., 96 141.}	3.44618
3	R0	{2.32253, 12.836}	{2.94849, 16.5051}	{{2.65918, 14.5488}, {3., 20.}}	{27.2774, 28.5844}	inf	1753.	2570.72	{2311.99, 2858.1}	8.05148	0.0375145	newly	2684.	1690.5	{1276., 2056.}	37.0156	0.209052	Reff	2.19238	2.85961	{2.57272, 2.92286}	30.604	ar	98 997.	96 511.4	{94 805., 97 442.}	2.51076
4	R0	{1.79075, 11.2058}	{2.99729, 19.8724}	{{3., 19.8511}, {3., 20.}}	{67.3762, 77.3402}	inf	2803.	6109.08	{5740.56, 6504.78}	50.4699	0.176873	newly	3933.	2919.69	{2756., 3107.}	25.7643	0.129867	Reff	1.61667	2.72643	{2.71526, 2.76425}	68.6446	ar	96 920.	97 726.6	{96 914., 98 305.}	0.867654
5	R0	{2.26952, 9.62371}	{2.88547, 15.6499}	{{2.26588, 14.3335}, {3., 20.}}	{29.0939, 62.6182}	inf	1270.	2127.61	{1617.65, 2716.28}	20.1529	0.086755	newly	3231.	1471.67	{947., 1911.}	54.4516	0.355235	Reff	2.17415	2.79257	{2.20947, 2.9304}	30.141	ar	98 881.	94 795.	{88 827., 96 588.}	4.1322
6	R0	{1.57191, 6.89004}	{2.94463, 16.5046}	{{2.84105, 15.1864}, {3., 20.}}	{87.3282, 139.543}	inf	1343.	3607.43	{3080.35, 4077.53}	83.584	0.260144	newly	3690.	2300.63	{1609., 2854.}	37.6523	0.218282	Reff	1.49482	2.73186	{2.47979, 2.83564}	82.7549	ar	95 046.	95 571.7	{94 295., 96 744.}	1.17905
7	R0	{1.99226, 17.2339}	{2.11644, 20.}	{{2.10316, 20.}, {2.12436, 20.}}	{6.23289, 16.0503}	inf	7484.	8082.77	{7973.19, 8137.16}	39.3185	0.216958	newly	6848.	3253.14	{3194., 3303.}	52.495	0.323298	Reff	1.39466	1.81865	{1.81025, 1.82828}	30.4008	ar	97 992.	89 754.6	{89 315., 90 092.}	8.40621
8	R0	{2.63369, 15.1879}	{2.98925, 20.}	{{2.95699, 20.}, {3., 20.}}	{13.5004, 31.6838}	inf	3695.	5203.29	{4862.84, 5347.87}	12.872	0.0601304	newly	4729.	2498.06	{2353., 2601.}	47.1757	0.277519	Reff	2.31741	2.78205	{2.75428, 2.80503}	20.0498	ar	99 451.	97 009.6	{96 657., 97 236.}	2.45486
9	R0	{2.53271, 9.90552}	{2.28978, 17.0291}	{{1.57118, 11.4986}, {3., 20.}}	{21.2011, 73.7803}	inf	823.	1021.46	{390.256, 1596.34}	32.6891	0.190043	newly	2227.	560.18	{288., 863.}	74.846	0.644098	Reff	2.46668	2.2417	{1.56758, 2.92896}	20.8796	ar	99 355.	84 358.2	{65 194., 96 849.}	15.0941
10	R0	{2.48791, 18.9003}	{2.79804, 20.}	{{2.73367, 20.}, {2.86491, 20.}}	{12.4656, 5.81843}	inf	5017.	6925.55	{6597.18, 7134.14}	17.6412	0.0845521	newly	4646.	3152.26	{3002., 3272.}	32.1511	0.168698	Reff	2.05964	2.50452	{2.43598, 2.56994}	21.5997	ar	99 291.	96 889.7	{95 680., 97 791.}	2.41844
11	R0	{2.84414, 18.4765}	{2.20797, 17.2769}	{{2.20316, 17.2471}, {2.2116, 17.2887}}	{22.3677, 6.65739}	inf	3971.	3457.53	{3386.96, 3487.8}	46.2281	0.269545	newly	4035.	1759.31	{1706., 1822.}	56.3988	0.360846	Reff	2.47881	2.10967	{2.11109, 2.11111}	14.892	ar	99 654.	90 337.5	{90 089., 90 634.}	9.3489
12	R0	{2.29369, 14.622}	{2.86047, 20.}	{{2.77867, 20.}, {2.90193, 20.}}	{24.7104, 36.7802}	inf	4819.	7143.77	{6956.08, 7226.29}	9.57255	0.043757	newly	5911.	3283.18	{3144., 3380.}	44.4564	0.255541	Reff	1.91186	2.55273	{2.50137, 2.58388}	33.5206	ar	98 982.	97 619.5	{96 207., 98 220.}	1.37653
13	R0	{2.73413, 16.7529}	{2.7766, 20.}	{{2.75225, 20.}, {2.79746, 20.}}	{1.55331, 19.3823}	inf	4263.	5110.41	{5048.17, 5174.06}	24.4022	0.121525	newly	4789.	2372.69	{2298., 2434.}	50.4554	0.305124	Reff	2.35852	2.59301	{2.57333, 2.61261}	9.94227	ar	99 543.	96 782.6	{96 480., 97 031.}	2.77309
14	R0	{1.79554, 16.5943}	{1.91385, 20.}	{{1.89758, 20.}, {1.93575, 20.}}	{6.5891, 20.5233}	inf	6015.	5270.18	{5243.33, 5294.26}	47.7424	0.281854	newly	5421.	2089.32	{2031., 2145.}	61.4588	0.414159	Reff	1.3756	1.71703	{1.70056, 1.74007}	24.8209	ar	96 821.	82 733.8	{82 013., 83 597.}	14.5497
15	R0	{1.91429, 13.7771}	{1.89132, 20.}	{{1.88075, 20.}, {1.91597, 20.}}	{1.23327, 45.1684}	inf	5411.	3841.02	{3832.25, 3853.76}	55.7843	0.354432	newly	6031.	1527.46	{1486., 1562.}	74.6732	0.596519	Reff	1.53354	1.75305	{1.74199, 1.77822}	14.3141	ar	97 541.	81 518.4	{80 768., 82 678.}	16.4265
16	R0	{1.42094, 10.5871}	{2.14179, 17.0167}	{{2.11677, 16.6661}, {2.15435, 17.1804}}	{50.7306, 60.7301}	inf	3733.	5922.81	{5746.73, 6022.53}	5.49518	0.0231521	newly	4920.	2939.79	{2859., 3011.}	40.2482	0.223731	Reff	1.20628	1.92183	{1.90413, 1.93059}	59.3188	ar	93 044.	87 123.9	{86 401., 87 642.}	6.36269
17	R0	{1.45515, 6.16466}	{2.78768, 16.2057}	{{2.09172, 13.2387}, {3., 20.}}	{91.5737, 162.881}	inf	733.	1823.83	{1217., 2387.33}	88.8958	0.267026	newly	2188.	1169.45	{820., 1649.}	46.5516	0.292214	Reff	1.41733	2.65336	{2.05298, 2.92961}	87.2083	ar	93 254.	93 398.1	{85 009., 96 858.}	4.08144
18	R0	{2.85669, 13.2416}	{2.59671, 18.4683}	{{1.89571, 13.6157}, {3., 20.}}	{13.8174, 42.0437}	inf	1540.	1775.85	{1104.86, 2312.02}	29.2323	0.167829	newly	2807.	910.06	{556., 1150.}	67.5789	0.506466	Reff	2.71777	2.52429	{1.82664, 2.92704}	14.0245	ar	99 644.	90 549.6	{78 739., 96 163.}	9.12693
19	R0	{2.56417, 17.9558}	{2.23637, 20.}	{{2.23513, 20.}, {2.24497, 20.}}	{12.784, 11.3846}	inf	4153.	4194.1	{3831.6, 4289.61}	37.4388	0.204192	newly	4054.	1785.52	{1629., 1869.}	55.9566	0.356657	Reff	2.21442	2.11285	{2.11012, 2.13019}	4.58673	ar	99 389.	91 063.8	{90 701., 91 440.}	8.37634
20	R0	{2.27517, 11.1864}	{2.91514, 17.1054}	{{2.82459, 15.1838}, {3., 20.}}	{28.7637, 52.9148}	inf	2051.	3354.25	{2860.73, 3938.95}	13.8059	0.056933	newly	3692.	2012.67	{1557., 2767.}	45.4856	0.27583	Reff	2.12451	2.69221	{2.46291, 2.84244}	27.2572	ar	98 894.	95 212.7	{94 138., 96 960.}	3.7225
21	R0	{2.35735, 10.4735}	{2.87962, 15.9786}	{{2.78486, 14.7794}, {3., 19.4916}}	{24.4503, 52.5751}	inf	1087.	1791.82	{1449.29, 2220.35}	19.1772	0.0785459	newly	2417.	1217.21	{783., 1690.}	49.6396	0.318023	Reff	2.2751	2.76189	{2.48642, 2.93316}	23.4783	ar	99 036.	94 665.6	{93 697., 96 510.}	4.41292
22	R0	{1.79551, 11.9179}	{2.95037, 20.}	{{2.91903, 20.}, {3., 20.}}	{64.3195, 67.8148}	inf	4439.	8904.34	{8548.6, 9086.66}	27.4598	0.105214	newly	5958.	4099.7	{3927., 4213.}	31.19	0.162535	Reff	1.5037	2.58706	{2.55722, 2.62702}	72.0458	ar	97 033.	99 404.7	{98 860., 100 399.}	2.44424
23	R0	{1.23545, 6.31687}	{3., 20.}	{{3., 20.}, {3., 20.}}	{142.827, 216.612}	inf	2814.	10 103.7	{9516.53, 10 427.9}	151.962	0.401094	newly	6598.	4613.28	{4397., 4795.}	30.0806	0.155641	Reff	1.0892	2.56046	{2.54123, 2.59335}	135.078	ar	88 739.	97 748.2	{97 271., 98 400.}	10.1524
24	R0	{2.24089, 10.1789}	{2.40435, 17.8404}	{{1.55894, 9.37959}, {3., 20.}}	{20.0279, 77.802}	inf	976.	1208.76	{532.664, 1953.48}	30.7219	0.163793	newly	2027.	618.33	{385., 991.}	69.4953	0.540234	Reff	2.17337	2.35683	{1.52916, 2.92265}	20.4829	ar	98 868.	86 700.6	{63 329., 95 949.}	12.3067
25	R0	{1.57298, 10.2607}	{2.09411, 15.9501}	{{2.07343, 15.8255}, {2.14701, 16.4503}}	{33.1303, 55.5508}	inf	2641.	3888.24	{3863.94, 4118.82}	3.7427	0.0186449	newly	3941.	2104.78	{2017., 2186.}	46.5927	0.27261	Reff	1.41523	1.95301	{1.93193, 2.00215}	37.9998	ar	94 957.	84 966.3	{84 665., 86 428.}	10.5213
26	R0	{1.2154, 8.2576}	{2.15549, 17.0239}	{{2.14972, 16.9449}, {2.15762, 17.1072}}	{77.3481, 106.16}	inf	4010.	7892.03	{7843.91, 7928.83}	33.9903	0.127069	newly	6408.	3851.04	{3770., 3925.}	39.9026	0.221199	Reff	0.993966	1.85894	{1.85573, 1.86105}	87.0227	ar	88 421.	88 833.9	{88 552., 89 118.}	0.474797
27	R0	{2.22841, 18.7983}	{2.1735, 17.0735}	{{2.169, 16.9857}, {2.17641, 17.1933}}	{2.46421, 9.17548}	inf	7582.	7277.16	{7209.54, 7314.63}	46.2345	0.269502	newly	6511.	3590.42	{3518., 3683.}	44.8561	0.258566	Reff	1.58429	1.92714	{1.92381, 1.9296}	21.641	ar	98 756.	91 551.7	{91 197., 91 900.}	7.29501
28	R0	{1.27778, 5.36767}	{2.95581, 16.7592}	{{2.87241, 14.6095}, {3., 20.}}	{131.324, 212.225}	inf	966.	2827.1	{2398.16, 2977.05}	126.203	0.350905	newly	2971.	1817.3	{1350., 2166.}	38.832	0.22446	Reff	1.23118	2.84682	{2.6216, 2.92001}	131.227	ar	89 843.	96 328.9	{95 117., 97 626.}	7.95959
29	R0	{2.38042, 15.8901}	{2.96084, 20.}	{{2.78492, 20.}, {3., 20.}}	{24.3831, 25.8645}	inf	3337.	5327.74	{4709.89, 5694.96}	5.15544	0.0224716	newly	3642.	2537.73	{2203., 2719.}	30.3204	0.15812	Reff	2.11469	2.73433	{2.57018, 2.7742}	29.3015	ar	99 112.	95 928.3	{95 689., 96 299.}	3.2122
30	R0	{2.53188, 12.7607}	{2.32837, 16.3228}	{{1.58449, 8.85704}, {3., 20.}}	{20.6755, 36.8817}	inf	1016.	1073.9	{429.373, 1630.27}	32.6328	0.19686	newly	1649.	607.47	{336., 866.}	63.1613	0.467281	Reff	2.45506	2.25947	{1.52966, 2.92901}	20.234	ar	99 317.	84 860.5	{65 817., 96 768.}	14.5559
31	R0	{1.24482, 10.5471}	{2.28785, 20.}	{{2.28627, 20.}, {2.2889, 20.}}	{83.7898, 89.6256}	inf	5483.	13 814.6	{13 727.3, 13 878.5}	62.1818	0.209998	newly	6713.	5469.1	{5380., 5547.}	18.5297	0.0890332	Reff	0.914831	1.7572	{1.75511, 1.75905}	92.079	ar	89 097.	96 227.	{95 930., 96 566.}	8.0025
32	R0	{2.30446, 17.9607}	{3., 20.}	{{3., 20.}, {3., 20.}}	{30.1823, 11.3542}	inf	5816.	9894.99	{9772.04, 10 001.2}	0.921187	0.00398026	newly	5244.	4533.26	{4432., 4633.}	13.5534	0.0633126	Reff	1.82382	2.57128	{2.56713, 2.57602}	40.9836	ar	99 028.	96 981.9	{96 750., 97 167.}	2.06614
33	R0	{2.31579, 11.8236}	{2.98576, 15.8081}	{{3., 15.44}, {3., 16.1131}}	{29.506, 34.74}	inf	3092.	4911.35	{4576.26, 5231.94}	6.72334	0.0301373	newly	5187.	3228.35	{2944., 3493.}	37.7607	0.206921	Reff	2.08296	2.7555	{2.75589, 2.78262}	32.7538	ar	99 012.	96 571.9	{96 476., 97 162.}	2.46449
34	R0	{1.6678, 10.6744}	{3., 20.}	{{3., 20.}, {3., 20.}}	{79.8777, 87.3642}	inf	4713.	11 477.4	{10 547.8, 11 642.}	61.064	0.206768	newly	6918.	5160.2	{4834., 5285.}	25.4091	0.127506	Reff	1.37288	2.49404	{2.48666, 2.54069}	81.6645	ar	95 809.	97 250.8	{97 080., 97 403.}	1.50483
35	R0	{1.65476, 7.07559}	{2.88538, 15.9478}	{{2.54134, 14.7826}, {3., 19.515}}	{74.3686, 125.392}	inf	893.	2018.9	{1495.72, 2586.49}	62.1752	0.20253	newly	2514.	1363.49	{896., 1752.}	45.7641	0.282427	Reff	1.60409	2.77901	{2.41179, 2.93306}	73.2451	ar	95 746.	94 791.1	{92 345., 96 486.}	1.69345
36	R0	{2.71807, 18.5959}	{2.61327, 19.6339}	{{2.59064, 20.}, {2.71698, 20.}}	{3.88488, 8.31101}	inf	3397.	4224.22	{4009.77, 4702.24}	20.2977	0.103862	newly	3163.	1947.39	{1787., 2132.}	38.4322	0.213328	Reff	2.4286	2.44902	{2.43813, 2.53466}	4.04044	ar	99 540.	93 179.8	{93 391., 94 519.}	6.38959
37	R0	{2.6202, 12.7552}	{1.99987, 17.5717}	{{1.68038, 15.5005}, {2.11861, 20.}}	{23.6747, 38.6116}	inf	1248.	1150.41	{906.556, 1348.14}	37.2391	0.209133	newly	2178.	563.93	{414., 705.}	74.1079	0.596745	Reff	2.51914	1.95622	{1.65773, 2.08258}	22.3459	ar	99 426.	82 005.9	{71 718., 85 741.}	17.5207
38	R0	{1.47672, 9.81651}	{2.13764, 16.5287}	{{2.13567, 16.5077}, {2.13817, 16.577}}	{44.7558, 68.3765}	inf	4210.	5888.32	{5870.51, 5915.24}	8.73648	0.0397159	newly	6266.	3021.3	{2959., 3078.}	51.7826	0.316847	Reff	1.23	1.91882	{1.91662, 1.91931}	56.0013	ar	93 815.	87 139.6	{86 837., 87 382.}	7.11553
39	R0	{1.90004, 12.3313}	{2.81092, 20.}	{{2.75924, 20.}, {2.85647, 20.}}	{47.9403, 62.1889}	inf	3615.	7286.31	{6895.28, 7650.08}	30.6961	0.115857	newly	4980.	3315.46	{3088., 3512.}	33.4245	0.177195	Reff	1.65284	2.50837	{2.48876, 2.52164}	51.761	ar	97 413.	97 168.8	{96 343., 98 001.}	0.685483
40	R0	{2.64218, 18.5237}	{3., 20.}	{{3., 20.}, {3., 20.}}	{13.5426, 7.96979}	inf	6412.	10 045.3	{9178.12, 10 366.4}	7.9422	0.0365261	newly	6088.	4578.54	{4228., 4761.}	24.794	0.124195	Reff	2.0572	2.547	{2.52073, 2.61439}	23.809	ar	99 474.	97 801.2	{97 404., 98 021.}	1.68168
41	R0	{1.6945, 7.17996}	{2.15425, 16.8144}	{{1.50886, 9.2196}, {2.92124, 20.}}	{32.2264, 134.185}	inf	498.	790.829	{303.479, 1314.94}	50.2496	0.198252	newly	1333.	415.	{180., 656.}	68.8672	0.551274	Reff	1.66739	2.11847	{1.49088, 2.87432}	32.2316	ar	96 332.	81 048.7	{61 134., 95 371.}	15.9309
42	R0	{1.84559, 18.0074}	{2.26259, 20.}	{{2.23877, 20.}, {2.28099, 20.}}	{22.5944, 11.0655}	inf	7319.	10 651.	{10 290.4, 10 830.6}	18.583	0.0893697	newly	6151.	4313.57	{4161., 4424.}	29.8721	0.15423	Reff	1.29117	1.87637	{1.86106, 1.88715}	45.323	ar	97 358.	95 113.6	{94 386., 95 855.}	2.3053
43	R0	{2.24965, 17.0361}	{2.80725, 20.}	{{2.73385, 20.}, {2.86136, 20.}}	{24.786, 17.3978}	inf	4414.	6681.37	{6146.52, 7305.69}	7.60529	0.0348176	newly	4281.	3050.9	{2814., 3309.}	28.7339	0.147996	Reff	1.90728	2.5277	{2.46883, 2.59107}	32.5291	ar	98 770.	97 014.9	{95 970., 97 907.}	1.777
44	R0	{2.54324, 15.7967}	{2.91393, 20.}	{{2.87231, 20.}, {2.94542, 20.}}	{14.5755, 26.6087}	inf	4842.	6740.2	{6528.78, 6809.81}	12.2485	0.0568019	newly	5449.	3142.15	{3022., 3245.}	42.3353	0.239226	Reff	2.13836	2.64707	{2.61768, 2.6712}	23.79	ar	99 354.	97 264.9	{96 794., 97 500.}	2.10268
45	R0	{2.54943, 14.8032}	{3., 20.}	{{3., 20.}, {3., 20.}}	{17.6734, 35.1059}	inf	3565.	5442.2	{5068.75, 5564.71}	3.38719	0.0153869	newly	4552.	2614.53	{2451., 2716.}	42.563	0.241224	Reff	2.25387	2.77966	{2.77245, 2.79766}	23.328	ar	99 380.	96 960.9	{96 699., 97 212.}	2.43416
46	R0	{2.37755, 12.6461}	{2.95869, 16.0919}	{{2.85401, 14.3752}, {3., 20.}}	{24.5903, 27.248}	inf	1629.	2417.9	{2001.06, 2750.38}	10.5247	0.0497078	newly	2717.	1641.94	{1222., 1990.}	39.5679	0.226667	Reff	2.25026	2.86033	{2.57442, 2.91621}	27.1434	ar	99 096.	96 059.6	{94 872., 97 255.}	3.06411
47	R0	{1.69279, 11.6636}	{2.21352, 17.4574}	{{2.17172, 17.426}, {2.23195, 17.5675}}	{30.7619, 49.6741}	inf	3683.	4157.33	{3983.88, 4217.28}	25.8546	0.130023	newly	4864.	2071.39	{2012., 2126.}	57.4139	0.370861	Reff	1.4668	2.0736	{2.0449, 2.09005}	41.3686	ar	96 214.	89 342.1	{88 683., 89 764.}	7.14228
48	R0	{1.82402, 10.0038}	{2.97845, 19.87}	{{3., 20.}, {3., 20.}}	{63.2904, 98.6248}	inf	1751.	4159.78	{3651.62, 4529.16}	60.1834	0.202893	newly	3183.	2031.48	{1762., 2209.}	36.1772	0.197031	Reff	1.71321	2.81833	{2.8268, 2.86098}	64.5058	ar	97 076.	96 837.3	{96 703., 97 299.}	0.408587
49	R0	{1.6424, 11.1667}	{3., 20.}	{{3., 20.}, {3., 20.}}	{82.6595, 79.1039}	inf	5534.	13 381.	{13 043.8, 14 022.3}	50.8737	0.178463	newly	7611.	5913.29	{5752., 6122.}	22.306	0.109734	Reff	1.28365	2.41616	{2.38307, 2.43184}	88.2259	ar	95 546.	98 157.1	{97 838., 98 474.}	2.73282
50	R0	{2.05638, 14.3048}	{3., 20.}	{{3., 20.}, {3., 20.}}	{45.8874, 39.8132}	inf	4454.	8539.47	{7971.23, 8854.38}	23.278	0.0905537	newly	5024.	3971.37	{3715., 4131.}	20.952	0.102428	Reff	1.73898	2.64579	{2.6283, 2.67415}	52.146	ar	98 194.	97 664.5	{97 360., 97 942.}	0.539188
